# Inflammatory bowel disease through the lens of microbe-host interactions: immunomodulation, metabolic effects, and genetic susceptibility in microbiota dysbiosis

**DOI:** 10.3389/fcimb.2026.1745929

**Published:** 2026-02-16

**Authors:** Qinghua Zou, Wumiao Zhang, Hua Xie, Shuyan Ying, Xueliang Zeng, Yihang Yao, Dingcheng Zeng, Jiulong Wang, Cheng Zhang, Fan Meng

**Affiliations:** 1 The First Clinical Medical College of Gannan Medical University, Ganzhou, Jiangxi, China; 2 The First Affiliated Hospital of Gannan Medical University, Ganzhou, Jiangxi, China; 3 The Yuebei People’s Hospital, Shaoguan, Guangdong, China; 4 The Fifth Affiliated Hospital of Southern Medical University, Guangzhou, China

**Keywords:** dysbiosis of microbial community, gene, immune system, inflammatory bowel disease, metabolites

## Abstract

Inflammatory bowel disease (IBD), including ulcerative colitis, Crohn’s disease, and inflammatory bowel disease-unclassified, is a complex intestinal disease influenced by microbial factors, genetic and environmental. IBD has become a global disease with an increasing prevalence, endangering human health worldwide. Through its interactions with host immunity, bacterial metabolites, and genetic components, the intestinal microbiome plays a crucial role in initiating and advancing IBD. Treatment for IBD includes not only corticosteroids, aminosalicylates, antibiotics, TNF-α, α4β7 integrins, IL-12/23 antibodies, and small molecule antibodies, but also complementary and alternative medical therapies such as probiotics and prebiotics. This review primarily explores the relationship between dysbiosis of the microbiota and IBD, including the immune system, metabolites, and genetics related to microorganisms, to provide a deeper and more systematic understanding of the mechanisms linking microbial imbalance to IBD.

## 1 Introduction

The gut microbiota includes viruses, bacteria, fungi, and archaea (Sorboni et al., 2022), with the gastrointestinal tract harboring over 10^14^ microbial cells (Thursby and Juge, 2017). Microbial dysbiosis generally refers to changes in the quantity or type of microbial communities, resulting in corresponding changes in the body. At present, studies have found that intestinal flora imbalance may lead to type 2 diabetes (Iatcu et al., 2021), Alzheimer’s disease (Manfredi et al., 2025), cardiovascular disease (Tang et al., 2017), rheumatoid arthritis (Cai et al., 2025), post-COVID-19 syndrome (Lau et al., 2025), colorectal cancer (Anderson and Sears, 2023), pancreatic duct adenocarcinoma (Fanijavadi and Jensen, 2025), IBD (Qiu et al., 2022)and other diseases. The prevalence of IBD is steadily rising. In the United States, between approximately 2.4 and 2.7 million people have been diagnosed with IBD (Lewis et al., 2023), while the number in Europe exceeds 3.2 million (Ananthakrishnan et al., 2020). The epidemiological progression of IBD occurs through four distinct phases: emergence, acceleration, compounding prevalence, and equilibrium (Kaplan and Windsor, 2021). As of 2020, developing nations remained in the emergence phase, while the western region was in the stage of compounding prevalence.

IBD is an idiopathic chronic gastrointestinal inflammatory disease, characterized by a multifactorial pathogenesis involving genetic predisposition, immune dysregulation, and gut microbial dysbiosis (Manichanh et al., 2012). IBD includes ulcerative colitis (UC), Crohn’s disease (CD), and other unclassified IBD. CD is characterized by discontinuous skip lesions affecting any segment of the gastrointestinal tract, featuring chronic transmural inflammation with a high recurrence tendency. Patients with this disorder frequently experience persistent abdominal discomfort, chronic diarrhea episodes, potential intestinal blockage, and the development of perianal lesions (Roda et al., 2020). Differing from CD, UC demonstrates a continuous pattern of superficial inflammation restricted to the colon, frequently leading to mucosal erosion, ulcer formation, chronic diarrheal episodes, and bloody rectal discharge (Gros and Kaplan, 2023). The pathogenic role of gut microbiota in IBD development may involve multiple mechanisms, including dysregulated immune activation, compromised intestinal barrier integrity, metabolic dysbiosis, and complex gene-microbiota interactions.

## 2 Mechanisms related to dysbiosis promoting IBD

### 2.1 From microbial chaos to immune disruption: pathogenic cascade reaction of microbial imbalance in IBD

The integumentary system and mucosal surfaces serve as primary physical barriers within the gastrointestinal tract, effectively excluding pathogenic microorganisms including bacteria, viruses and fungi from host invasion (Di Tommaso et al., 2021). When pathogenic microorganisms penetrate these physical barriers, they activate the innate immune system, leading to antimicrobial peptide (AMP) production and the mobilization of various immune cells to eliminate the invading pathogens (Guryanova and Ovchinnikova, 2022).

The intestinal barrier function not only relies on various immune cell populations including macrophages, dendritic cells (DCs), innate lymphocytes (ILCs), and T and B cells in the adaptive immune system, but also involves the important involvement of non-immune intestinal epithelial cells (IECs) (Ghosh et al., 2021). These cellular populations are essential for maintaining host-microbiota homeostasis through the coordination of complex signaling networks that modulate mucosal immunity and mediate microbial-derived communication. Studies have found that the spleen and peripheral lymph nodes of germ-free mice are underdeveloped, and mesenteric lymph nodes are usually absent. Furthermore, commensal microbiota modulate the population dynamics and functional activity of T cells, B cells, and innate immune cells ( (Kuhn and Stappenbeck, 2013).

Serving as fundamental elements of innate immunity, pattern recognition receptors (PRRs) assemble into a sophisticated molecular detection network through multiple protein components. Functioning as key regulators, they maintain intestinal immune balance and microbial equilibrium by promptly recognizing microbial-derived pathogen associated molecular patterns (PAMPs) and host tissue-derived damage associated molecular patterns (DAMPs) (Burgueño and Abreu, 2020; Mehto et al., 2019). PRRs include RIG-I-like receptors (RLRs), Toll-like receptors (TLRs), C-type lectin receptors (CLRs), and NOD-like receptors (NLRs) (Takeuchi and Akira, 2010). Abnormal activation of PRRs can lead to immunodeficiency.

The activation of TLRs by symbiotic microbial communities can prevent intestinal damage and play a crucial role in maintaining intestinal homeostasis (Rakoff-Nahoum et al., 2004). TLR activation by bacterial products on intestinal epithelial cells stimulates epithelial proliferation, enhances luminal immunoglobulin A (IgA) secretion, and upregulates AMP expression. Dysregulation of this process can lead to chronic inflammation (Abreu, 2010). As the dominant immunoglobulin isotype within the intestinal lumen, IgA serves critical functions in shaping gut microbiota composition and maintaining intestinal equilibrium (Kawamoto et al., 2014). As the primary adaptor protein for most TLRs (excluding TLR3), MyD88 serves as an indispensable adaptor protein in TLR signaling pathways. MyD88-deficient mice demonstrate impaired T cell function and exacerbated colitis, highlighting its critical role in immune regulation. The MyD88-dependent signaling pathway facilitates the differentiation of antigen-specific inducible regulatory T cells (iTregs) within the intestinal mucosa, thereby orchestrating appropriate immune responses to commensal microbiota and maintaining inflammatory homeostasis (Wang et al., 2015). Meanwhile, MyD88 mediates T follicular regulatory (Tfr)/T follicular regulatory helper cells (Tfh) differentiation to orchestrate IgA responses against commensal microbiota in Peyer’s patches (PP).

As cytosolic pattern recognition receptors, NOD1 and NOD2 initiate signaling cascades that activate both nuclear factor κB (NF-κB) and mitogen-activated protein kinase (MAPK) pathways. NF-κB activation requires the adaptor molecule RIP2 (Park et al., 2007), whereas the MAPK pathway is mediated by caspase recruitment domain family member 9 (CARD9) ( (Liu et al., 2022). Hyperactivation of the NLR family pyrin domain-containing 3 (NLRP3) inflammasome impairs intestinal homeostasis by disrupting epithelial regeneration and compromising the gut-vascular barrier (Huang et al., 2019). Metabolites related to the microbiota, such as taurine, histamine, and spermine, can stimulate the formation of NLRP6 inflammasomes and induce the synthesis and secretion of downstream pro-inflammatory cytokines (Levy et al., 2015). NLRP10 exerts protective effects against inflammatory responses in the intestinal mucosa (Zheng et al., 2023).

Functioning in parallel to innate immunity, the adaptive immune response constitutes a second major immunological defense mechanism. At the cellular level, adaptive immune responses are coordinated by three principal lymphocyte populations: antibody-producing B cells, cytotoxic CD8^+^ T cells, and helper CD4^+^ T cells, which collectively mediate cellular and humoral immunity (Tomar and De, 2014).

T cells are roughly divided into pro-inflammatory and anti-inflammatory functional subsets based on their cytokine profiles and immunoregulatory properties. The cytotoxic capacity of pro-inflammatory CD8^+^ T cells underlies their clinical significance in tumor immunotherapy, metastasis control, and antiviral immunity (Zhang and Bevan, 2011). As master regulators of adaptive immunity, CD4^+^ T cells coordinate multiple immunological processes including inflammatory tone control, antibody-mediated protection, innate-adaptive crosstalk, and memory cell generation. The functional diversity of CD4^+^ T cells is reflected in their differentiation into multiple subsets (Tfh, T helper 1- Th1, Th2, Th9, Th17, Th22) and several types of Tregs, with each population exhibiting distinct cytokine signatures that determine their immunological roles (Hirahara and Nakayama, 2016).

**Figure 1:**
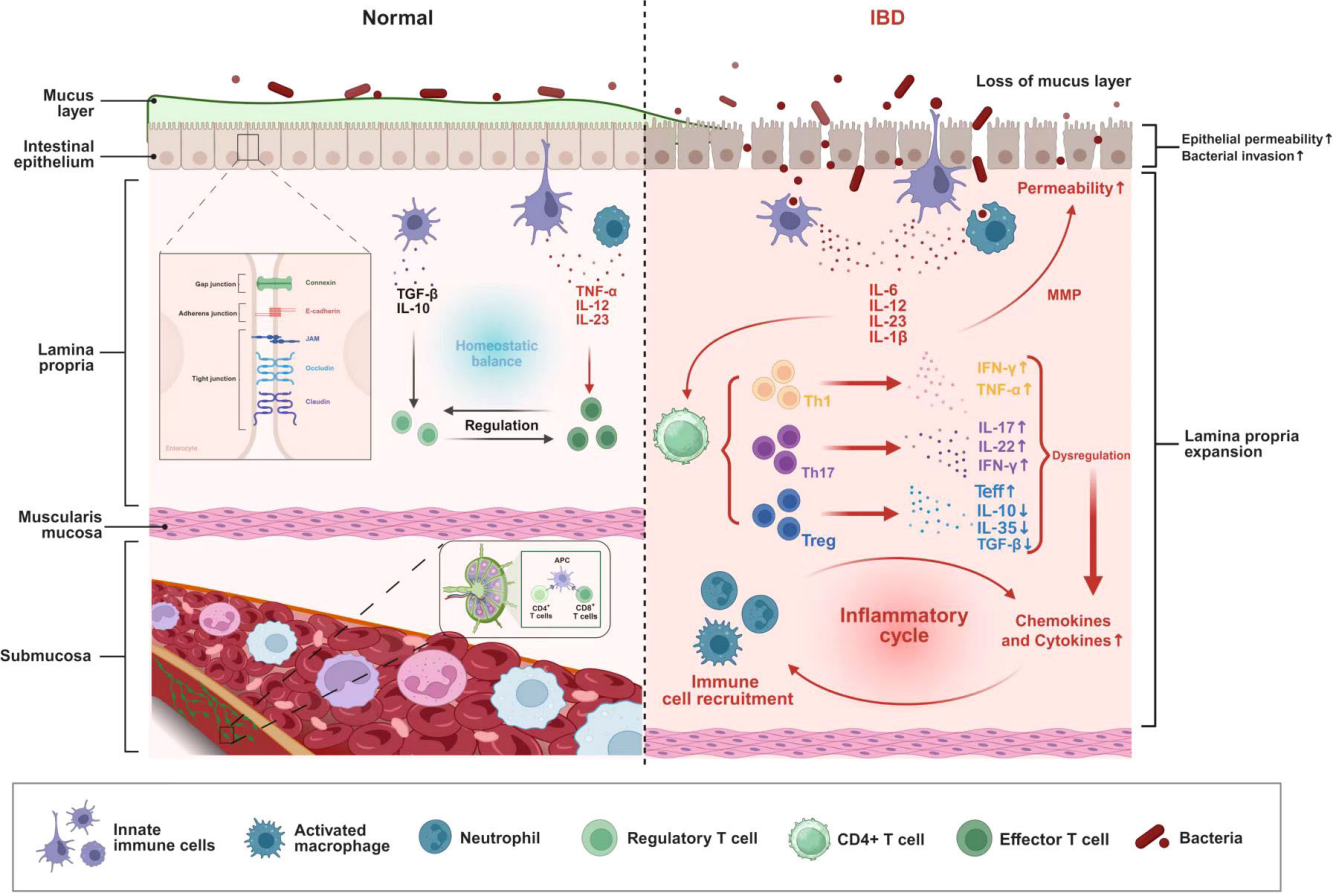
Impact of dysbiosis on immunity in IBD. Fimbriae-induced TLR2 IL-6, IL-23, IL-1β, and IL-12 promote the differentiation of Th17 and Th1 cells, thereby increasing the secretion of IFN-γ, TNF-α, IL-17, and IL-22. However, dysbiosis inhibits Treg cells, leading to reduced secretion of TGF-β, IL-10, and IL-35, as well as decreased apoptosis of Teff cells. These alterations not only cause damage to intestinal epithelial cells but also disrupt immune homeostasis. They interact with each other and ultimately contribute to the development of IBD.

Tregs, an anti-inflammatory CD4^+^ T cell subset, suppress excessive inflammation, promote immune tolerance, and maintain immune homeostasis to prevent autoimmunity (Sakaguchi et al., 2010). Microbial dysbiosis impairs Treg functionality while simultaneously driving the polarization of pro-inflammatory Th1 and Th17 subsets—characterized by increased production of interferon-gamma (IFN-γ), and interleukin-17 (IL-17)/IL-22 respectively—ultimately leading to the breakdown of immunological homeostasis (Neurath, 2014; Round and Mazmanian, 2009) (**Figure 1**). *Porphyromonas gingivalis* has been shown to worsen colitis via a tripartite mechanism involving gut dysbiosis, altered linoleic acid metabolism, and Th17/Treg cell imbalance (Jia et al., 2024).

Tregs can be classified into thymus-derived Tregs, also known as natural Treg cells (nTregs) and peripheral Tregs (pTregs) after thymic maturation. Unlike nTregs, iTregs are peripherally induced from naive CD4^+^ T cells through transforming growth factor-Beta (TGF-β) and IL-2 stimulation under *in vitro* conditions (Abbas et al., 2013). Functioning as the dominant control factor, the transcription factor Forkhead box protein P3 (FOXP3) orchestrates the entire genetic program responsible for Treg differentiation and immunosuppressive activity (Fontenot and Rudensky, 2005). Through their production of anti-inflammatory mediators (including TGF-β, IL-10 and IL-35) and cell-contact dependent suppression, Tregs modulate pathogenic inflammatory responses in a wide range of immune-mediated diseases (Mayne and Williams, 2013). Tregs induce apoptosis in effector T cells (Teffs) through multiple mechanisms, including competitive cytokine sequestration and direct cytotoxic interactions mediated by granzyme-perforin pathways (Hori et al., 2017) (**Figure 1**).

Patients with CD consistently demonstrate heightened Th1-type immune responses in both mucosal tissues and systemic circulation (Leppkes and Neurath, 2020). The commitment of naive CD4+ T cells to the Th1 lineage is triggered by IL-12-mediated signaling originating from activated antigen-presenting cells (APCs). Upon attaining maturity, Th1 effector cells secrete IFN-γ and TNF-α, which perform pleiotropic functions by acting upon innate immune cells—such as macrophages and neutrophils—as well as non-hematopoietic stromal cell populations (Caza and Landas, 2015). Cytokine-mediated Th17 polarization, induced by the combinatorial signaling of IL-6, IL-23, and IL-1β, generates pathogenic effector cells (Ghoreschi et al., 2010). These Th17 populations in CD patients acquire aberrant plasticity, evidenced by their concurrent production of IL-17 and IFN-γ (Annunziato et al., 2007) (**Figure 1**). In patients with IBD, peripheral blood shows significant expansion of Th17 cell populations, accompanied by increased concentrations of IL-17, IL-21, and IL-23 in both mucosal tissues and systemic circulation. The degree of IL-17 secretion by peripheral blood mononuclear cells (PBMCs) serves as an immunological biomarker that reflects disease severity in IBD, with higher cytokine production corresponding to more severe clinical manifestations (Raza and Shata, 2012).

A consequence of excessive IL-17 and IL-21 in the mucosa is the stimulation of myofibroblasts. This stimulation prompts them to release matrix metalloproteinases (MMPs), which ultimately degrade the extracellular matrix and harm epithelial cells (de Almeida et al., 2022). As a potent immunoregulator, IL-21 reinforces Th1 immunity by simultaneously boosting IFN-γ production and activating Th1-differentiation factors across adaptive (T cell) and innate (natural killer cell, NK cell) lymphocyte populations (Strengell et al., 2002). Furthermore, Th17-derived tumor necrosis factor-alpha (TNF-α) exists in distinct transmembrane and soluble isoforms, with membrane-bound TNF particularly activating TNFR2 signaling to exacerbate intestinal inflammation (Perrier et al., 2013).

### 2.2 Microbial metabolic disruption: how the by-products of dysbiosis disrupt intestinal homeostasis

The metabolites derived from the gut microbiota mainly include short-chain fatty acids (SCFAs: butyrate, acetate, propionate), trimethylamine, secondary bile acids (SBAs), lipopolysaccharides (LPS), imidazole propionate, and branched-chain amino acids, all of which have been linked to various diseases (Agus et al., 2021). Next, we will primarily explore the microbiota-derived metabolites related to IBD.

**Figure 2:**
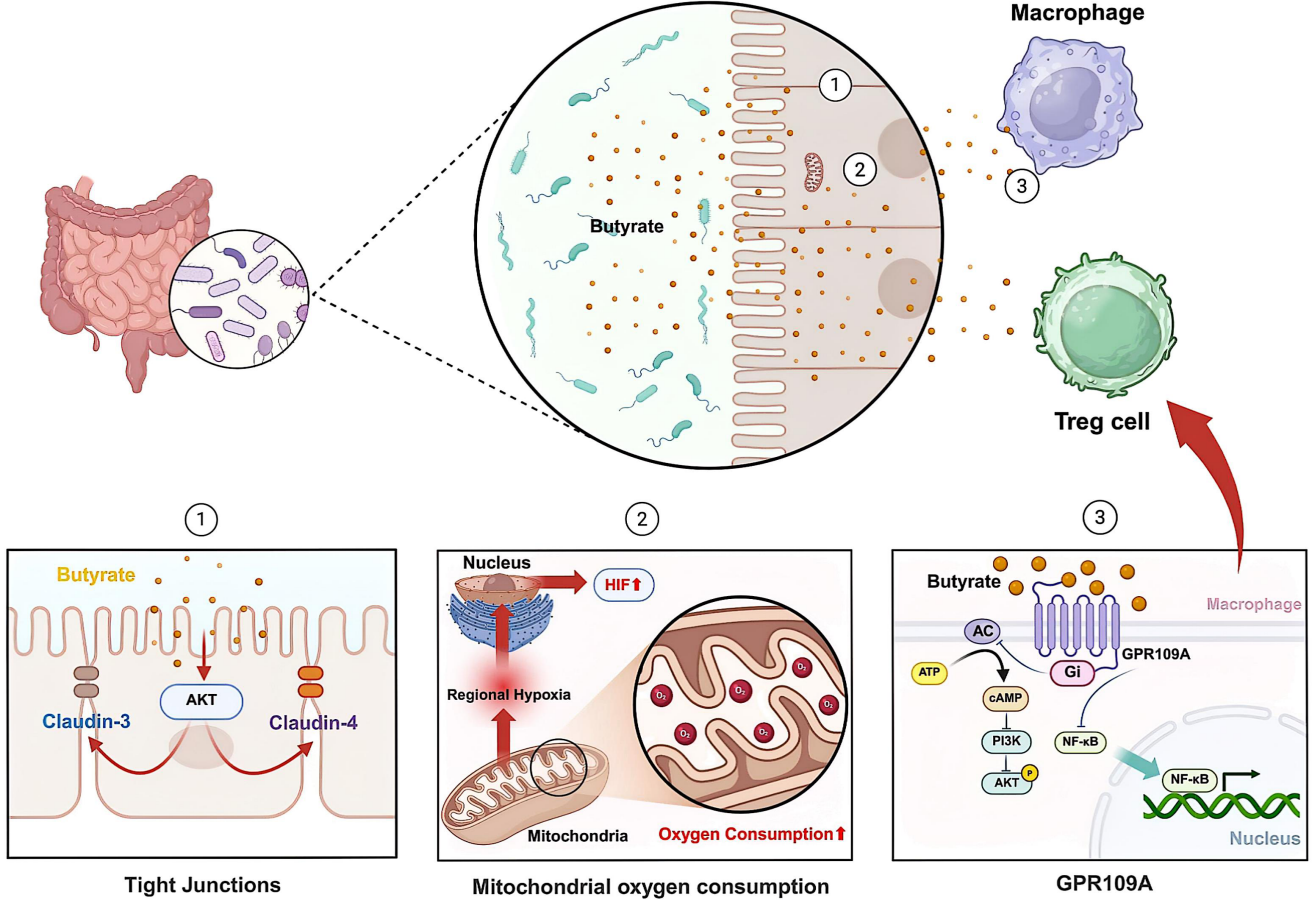
Role of butyrate in IBD. Butyrate not only enhances barrier function by activating Akt and upregulating the expression of claudin-3 and claudin-4, but also increases mitochondrial oxygen consumption in IECs, stabilizes HIF, and upregulates the expression of HIF target genes, thereby further strengthening barrier integrity. On the other hand, butyrate inhibits the phosphorylation of the AKT and NF-κB signaling pathways in macrophages in a GPR109A-dependent manner, which subsequently influences Treg cells.

The disruption of intestinal barrier function is a characteristic of IBD. IECs establish physical and biochemical barriers that segregate host tissues from the commensal microbiota, thereby maintaining intestinal homeostasis (Peterson and Artis, 2014).IECs include goblet cells that secrete mucins, nutrient-absorbing enterocytes, intestinal endocrine cells, Paneth cells that secrete AMPs, antigen-presenting (M) cells, and tuft cells mediating type 2 antiparasitic immunity (Soderholm and Pedicord, 2019). Intestinal barrier dysfunction in IBD arises from compromised tight junction integrity, featuring both loss of barrier-forming proteins (junctional adhesion molecule-A) and gain of pore-forming proteins that permit abnormal paracellular flux (Vetrano et al., 2008; Zeissig et al., 2007). SCFAs, derived from bacterial fermentation of dietary fiber, strengthen intestinal barrier integrity at physiological concentrations (Peng et al., 2007; Mariadason et al., 1997). Butyrate can promote AMP-activated protein kinase (AMPK) activity and accelerate tight junction assembly (Peng et al., 2009). Butyrate may activate the a-kinase transforming (AKT) mediated protein synthesis pathway, upregulating the expression of claudin-3 and claudin-4 (Yan and Ajuwon, 2017) (**Figure 2**). Claudins serve as the primary structural and functional components of tight junctions, mediating their paracellular barrier function (Tsukita et al., 2019; Günzel and Yu, 2013).

Through stimulating mitochondrial oxidative phosphorylation in IECs, butyrate induces pseudohypoxia that stabilizes hypoxia-inducible factor (HIF) and subsequently upregulates HIF-targeted genes, ultimately strengthening the epithelial barrier (Kelly et al., 2015) (**Figure 2**). Microbial metabolite butyrate can also weaken neutrophil function and improve mucosal inflammation in IBD (Li et al., 2021).

In the colonic mucosa, G protein-coupled receptor 109A (GPR109A) functions as a dual-affinity receptor that binds both microbial-derived butyrate and the essential nutrient niacin (vitamin B3), mediating their downstream signaling effects. Notably, niacin - also produced by gut microbiota - exerts anti-inflammatory effects. Through GPR109A-mediated signaling pathways, colonic macrophages and dendritic cells acquire enhanced anti-inflammatory capacity, enabling them to induce differentiation of Tregs and IL-10-producing T cells (Singh et al., 2014). The SCFA butyrate, acting through GPR109A receptors, downregulates pro-inflammatory signaling in macrophages by blocking AKT and NF-κB p65 phosphorylation events (Chen et al., 2018a) (**Figure 2**).

SCFAs not only participate in the intestinal barrier, but also regulate the production of IgA (Chen et al., 2020). There are two main mechanisms for the production of intestinal IgA: T cell-independent and T cell-dependent mechanisms (Tezuka and Ohteki, 2019). The gut microbiota predominantly enhances T cell-independent IgA generation (Tan et al., 2022). Functioning as a multifunctional immune mediator, IgA can defend against pathogens while simultaneously shaping the gut microbiota composition and maintaining immunological balance in the intestinal mucosa (Pietrzak et al., 2020). SCFA also inhibits histone deacetylase (HDAC) and activates mammalian target Of rapamycin (mTOR) to produce IgA induced Tregs (Smith et al., 2013). Both SCFA induced Tregs and IgA can prevent mucosal inflammation (McCoy et al., 2017).

Research has found that dysbiosis can contribute to the development of IBD by influencing bile acid metabolism (Duboc et al., 2013). The synthesis of SBAs—including lithocholic acid (LCA), deoxycholic acid (DCA), and ursodeoxycholic acid (UDCA)—occurs in the intestinal lumen through the sequential microbial transformation of primary bile acids, a process catalyzed by bacterial enzymes (di Gregorio et al., 2021). Activation of G protein- coupled bile acid receptor 1 (GPBAR1) by bile acids in intestinal stem cells initiates pro-regenerative signaling that promotes epithelial repair (Sorrentino et al., 2020). Bile acids regulate the immune system through their excitatory effects on the farnesoid X receptor (FXR) and GPBAR1, as well as their antagonistic effects on the retinoid-related orphan receptor γt (RORγt). These receptors induce macrophages and T helper cells to polarize towards anti-inflammatory phenotypes (M2 and Treg macrophages, respectively), upregulate IL-10 production, and inhibit DC, ILC3, and Th17 activation by downregulation of IL-1β, IL-6, IL-17 and TNF-α (Biagioli et al., 2021).

**Figure 3:**
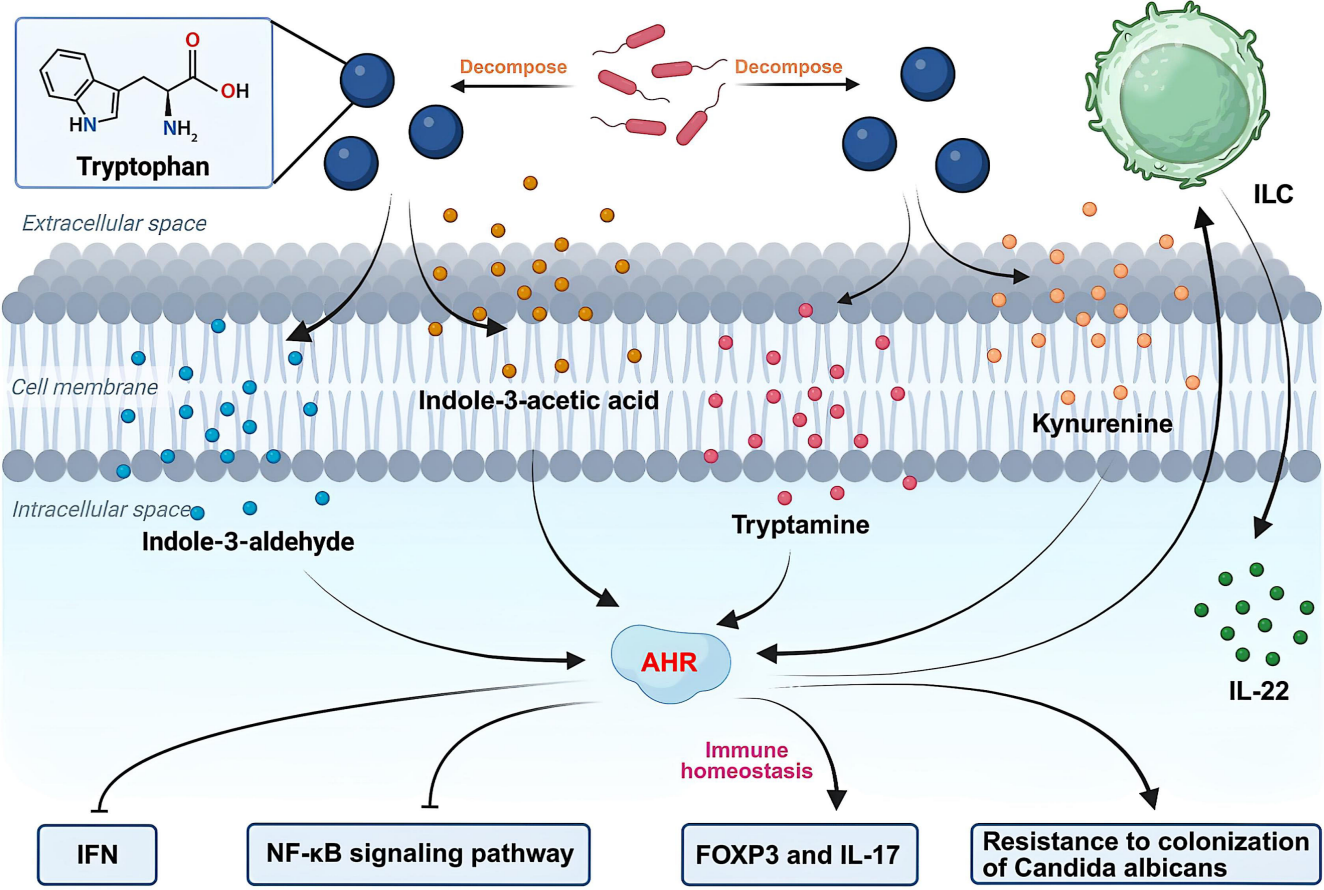
The involvement of tryptophan metabolites in IBD. Tryptophan metabolites, such as indole-3-acetic acid, indole-3-aldehyde, kynurenine, and tryptamine, can act as ligands for the aryl hydrocarbon receptor (AHR). The activation of AHR exerts multiple effects: it promotes the production of IL-22 by ILC, negatively regulates type I IFN and NF-κB signaling pathways, and enhances colonization resistance against *Candida albicans*.

Multiple tryptophan-derived metabolites—including indole-3-acetic acid, indole-3-aldehyde, kynurenine, and tryptamine —function as endogenous ligands for aryl hydrocarbon receptors (AHRs) (Jin et al., 2014; Schiering et al., 2017) (**Figure 3**). AHR activation is essential for intestinal barrier protection, primarily through its ability to stimulate ILC to secrete the epithelial-protective cytokine IL-22 (Lee et al., 2011; Zindl et al., 2013). Activation of AHR can inhibit inflammatory cytokines in the intestine through the IL-22-STAT3 pathway (Islam et al., 2017). AHR ligands may provide a method to inhibit endothelial activation and avoid sustained inflammatory responses to intestinal pathogens (Wiggins et al., 2023). Beyond its transcriptional activator functions, AHR demonstrates potent suppressive activity against type I IFN and NF-κB-mediated inflammatory signaling (Kimura et al., 2009; Yamada et al., 2016; Palrasu et al., 2025) (**Figure 3**). Microbiota-derived tryptophan metabolites balance mucosal immune responses by engaging the IL-22–AHR12 axis, thereby enabling the survival of polymicrobial communities while enhancing colonization resistance against *Candida albicans* and alleviating mucosal inflammation (Zelante et al., 2013). AHR can regulate immune homeostasis by modulating the expression of FOXP3 and IL-17 mRNA (Islam et al., 2017) (**Figure 3**).

Folic acid, a micronutrient produced by commensal microbes including *Bifidobacterium* and *Lactobacillus* species, serves as a critical cofactor in one-carbon metabolic pathways that ultimately produce S-adenosylmethionine (SAM) - the universal methyl donor for epigenetic modifications including DNA and histone methylation (Woo and Alenghat, 2022). Folate deficiency induces hyperhomocysteinemia that exacerbates intestinal Th17 cell responses (Wang et al., 2017), while folic acid supplementation demonstrates therapeutic potential in IBD. Indole-3-propionic acid (IPA), a metabolite derived from gut microbiota, promotes Th1/Th17 cell apoptosis by interacting with heat shock protein 70 (HSP70) (Gao et al., 2025). Recent groundbreaking research published in *Cell Metabolism* by Professor Hong Jie’s team at Renji Hospital, Shanghai Jiao Tong University School of Medicine revealed that microbiome-derived L-ornithine suppresses Th17-mediated inflammation and synergistically enhances the therapeutic efficacy of ustekinumab in CD (Wang et al., 2025). Compared with non-IBD populations, IBD has lower levels of trimethylamine-N-oxide (TMAO), which may provide a reference for IBD diagnosis and evaluation of disease activity (Wilson et al., 2015). Hydrogen sulfide and 5-aminosalicylic acid may also be related to IBD (Pitcher et al., 2000; Rowan et al., 2009), their exact mechanistic contributions require further elucidation.

### 2.3 Gene connections related to dysbiosis in microbial ecology and susceptibility to IBD

Numerous studies have shown a correlation between IBD and genetics, with a family history of IBD being an important risk factor for the disease (Farmer et al., 1980; Parkes and Jewell, 2001). Through genome-wide association studies (GWAS), over 200 genetic risk factors for IBD have been identified (Liu et al., 2015), including genes related to host microbiota mediated responses. The genes associated with IBD include *NOD2, IL23R, IRGM, CARD9, FUT2, XBP1, DOCK2* (Zhang et al., 2008; Beaudoin et al., 2013). Furthermore, genetic evidence implicates impaired intestinal barrier function in UC pathogenesis, with identified risk variants in *HNF4A*, *LAMB1*, *CDH1* and *GNA12* (Lees et al., 2011).

*NOD2* is closely associated with the CD phenotype (Hampe et al., 2002), and variations in the coding region of *NOD2* rich leucine repeat may affect the interaction between the host and bacterial lipopolysaccharides (Cho, 2001). Research has found that NOD2 can induce NF-κB activation in cells infected with *Pseudomonas aeruginosa, Campylobacter jejuni, Shigella flexneri*, and *Helicobacter pylori* (Mukherjee et al., 2019). With its distinctive domain organization—dual N-terminal CARD domains for signaling, a central NACHT domain for nucleotide binding, and a C-terminal LRR domain for ligand sensing—NOD2 serves as a crucial intracellular pattern recognition receptor (Werts et al., 2006). Under steady-state conditions, NOD2 exists in the cytoplasm in a self-inhibitory monomeric state. The NOD2 activation cascade involves two distinct structural events: first, NACHT domain-driven multimerization and conformational change; second, establishment of a CARD-mediated signaling complex with receptor-interacting serine-threonine kinase 2 (RIPK2). RIPK2 acts as a scaffold protein, providing an interface for the interaction of downstream signal transduction mediators (Hrdinka et al., 2018). RIPK2 mediates the formation of a ternary signaling complex through its interaction with transforming growth factor beta activated kinase 1 (TAK1) and associated binding proteins, including TAK1-binding protein 1 (TAB1), TAB2, and TAB3 (Wang et al., 2001). This cascade of molecular interactions leads to the formation of multi-component signalosomes that initiate downstream activation of both NF-κB and MAPK signaling cascades, culminating in the transcriptional upregulation of genes encoding pro-inflammatory cytokines and AMPs (Ogura et al., 2001; Caruso et al., 2014) (**Table 1**). NOD2 and gut symbiotic bacterial communities may maintain balance through feedback mechanisms, and mutations can disrupt this balance (Petnicki-Ocwieja et al., 2009). The most common *NOD2* susceptibility variants (SNP8, SNP12, and SNP13) correspond to rs2066844 (p.Arg702Trp), rs2066845 (p.Gly908Arg), and rs2066847 (p.Leu1007fs), respectively. The *NOD2* gene encodes intracellular receptors for bacterial peptidoglycan cell wall dipeptides, which form active oligomers upon stimulation and can trigger pro-inflammatory signaling cascades or stimulate autophagy by binding to ATG16L1. *NOD2* mutations lead to loss of pro-inflammatory signals, impaired autophagy, and reduced bacterial clearance, resulting in upregulation of alternative inflammatory pathways, including activation of IL-1β, IL-18, and NLRP3 inflammasomes (Bonen et al., 2003; Ashton et al., 2022). From this, it appears that the loss of *NOD2* can also lead to abnormal inflammatory reactions. Research has found that deficiency of the mouse Atg16L1 protein disrupts the recruitment of the Atg12–Atg5 protein conjugate to the isolation membrane, resulting in the loss of microtubule associated protein 1 light chain 3 and phosphatidylethanolamine conjugates, severely impairing the formation of autophagosomes (Saitoh et al., 2008), thereby promoting excessive production of IL-1 β and IL-6 (Plantinga et al., 2011).

**Table 1:** Mechanism of dysbiosis-associated genes in IBD pathogenesis.

Genotype	Mechanism	Related substances or reactions
*NOD2* (Hampe et al., 2002)	NF-κB (Mukherjee et al., 2019; Ogura et al., 2001)	pro-inflammatory cytokines, antimicrobial peptides
	MAPK (Caruso et al., 2014)	
	autophagosomes (Saitoh et al., 2008)	IL-1β, IL-6
*IL23R* (Duerr et al., 2006)	Th17 (Tesmer et al., 2008; Sewell and Kaser, 2022)	IL-17, IFN-γ, IL-22, GM-CSF
*IRGM* (Hunn et al., 2011)	NLRP3 (Mehto et al., 2019)	IL-1β
	inflammasome (Mehto et al., 2019)	p62
*CARD9* (Zhang et al., 2023)	IL-22 pathway (Sokol et al., 2013)	Th17、IFNγ、IL-6
*FUT2*	α1,2-fucosyltransferase (Rausch et al., 2011)	Alteration of the microbial community
	LPC (Tang et al., 2021)	Affecting susceptibility
*XBP1* (Kaser et al., 2008)	ER (Kaser et al., 2008)	Affecting the inflammatory response
*DOCK2* (Chen et al., 2018b)		

The *IL23R* gene, identified through GWAS as significantly associated with IBD, produces the IL-23 receptor that critically regulates inflammatory pathways and influences IBD risk (Duerr et al., 2006). The pro-inflammatory properties of IL-23 have become a major research focus, particularly its critical role in modulating Th17 cell biology (**Table 1**). As a specialized CD4+ T helper subset, Th17 cells constitutively reside in the intestinal lamina propria where they maintain baseline IL-17 production under homeostatic conditions (Tesmer et al., 2008). Histopathological analyses revealed markedly elevated Th17 cell infiltration in the inflamed mucosa of both CD and ulcerative colitis UC patients compared to healthy controls (Jiang et al., 2014). By promoting the differentiation, survival, and functional capacity of Th17 cells, IL-23 ultimately induces the release of pro-inflammatory mediators, including IL-17, IL-22, granulocyte-macrophage colony-stimulating factor (GM-CSF), and IFN-γ (Sewell and Kaser, 2022). Beyond its pro-inflammatory effects, IL-23 exhibits Treg-suppressive activity in the gut via molecular mechanisms that ultimately facilitate inflammatory responses and disease progression (Izcue et al., 2008). Tregs are a unique type of immune suppressive cell that participates in regulating many immune responses and plays important roles in physiological processes and diseases (Shao et al., 2021). Genetic studies have demonstrated that most documented variants in the IL-23R gene confer protection against IBD (Krause-Kyora et al., 2025). The protective effect of certain IL-23R polymorphisms (e.g., rs11209026, rs76418789, rs41313262) against IBD stems from their ability to compromise receptor function, stability, or signaling efficiency (Pastras et al., 2025).

The immune-related GTPase M (*IRGM*), a member of the interferon-inducible GTPase (IRG) family, serves as one of the most potent cell-autonomous defense systems against intracellular pathogens and plays a pivotal role in host immune defense (Hunn et al., 2011). *IRGM* can regulate the production of IL-1β through two mechanisms (Mehto et al., 2019). First, *IRGM* directly inhibits NLRP3 inflammasome activation by binding to the NACHT domain of NLRP3 (a supramolecular complex that activates caspase-1 (Sharma and de Alba, 2021)), thereby preventing NLRP3 oligomerization (**Table 1**). Concurrently, *IRGM* suppresses apoptosis-associated speck-like protein (ASC, including caspase recruitment domain (Fu and Wu, 2023)) aggregation, ultimately blocking inflammasome assembly. Second, IRGM promotes inflammasome clearance via p62-dependent selective autophagy, mediating the degradation of both NLRP3 and ASC (Shi et al., 2012) (**Table 1**).

In addition to the aforementioned genes, several additional genes contribute to IBD pathogenesis (**Table 1**). As a central signaling hub downstream of PRRs, *CARD9* coordinates mucosal defense by activating the IL-22-mediated repair pathway. This protective function is evidenced by the heightened colitis susceptibility observed in *CARD9*-deficient mice (Zhang et al., 2023; Lamas et al., 2016). Notably, *CARD9* knockout mice exhibit heightened susceptibility to experimental colitis, accompanied by defective secretion of key inflammatory mediators including Th17-associated cytokines, IL-6, and IFN-γ (Sokol et al., 2013). *FUT2* encodes an α1,2-fucosyltransferase that synthesizes ABO antigens in gut mucosa and secretions. It affects the composition, diversity, and structure of the microbial community (Rausch et al., 2011), and alters the functional status of the human intestinal mucosal surface microbiota (Tong et al., 2014). Intestinal FUT2 deficiency in the intestine can regulate the gut microbiota, promote LPC production, and ultimately increase susceptibility to IBD (Tang et al., 2021). Research suggests that *XBP1* may promote the occurrence of IBD through endoplasmic reticulum (ER) stress (Kaser et al., 2008), and *XBP1* guides the transcription of the core genome involved in ER function in all cell types (Acosta-Alvear et al., 2007). *DOCK2*, as an atypical guanine nucleotide exchange factor, contributes to the pathogenesis of multiple inflammatory disorders while paradoxically exerting protective effects during enteric bacterial infections through its regulation of small GTPase activity (Chen et al., 2018b).

## 3 Precision medicine in IBD: from pathogenic insights to tailored therapeutic regimens

IBD is a global disease for which there is currently no cure. Traditional medications for treating IBD include corticosteroids, aminosalicylates, immunomodulators, and antibiotics. At present, emerging therapeutic drugs include TNF-α, α4β7 integrin, IL-12/23 and IL-23 antibodies, as well as small molecule antibodies (Stallmach et al., 2023). The majority of IBD therapeutics have immunosuppressive properties, which can elevate susceptibility to infections and carcinogenesis (Villablanca et al., 2022). There is growing interest in developing new therapies with fewer side effects and higher treatment compliance. From this perspective, complementary and alternative medicine (CAM) is a treatment method that often uses natural compounds for medicinal purposes, demonstrating a promising complementary option in traditional medicine that allows for reducing drug dosage, frequency, or maintaining remission periods (Millstine et al., 2017).

A large number of promising nutritional supplements and natural compounds can be used for IBD treatment, which can be roughly divided into the following five categories. Polyphenols are a class of compounds composed of one or more phenyl rings combined with one or more hydroxyl moieties, which can alleviate gastrointestinal discomfort and have a positive impact on intestinal inflammation and gut microbiota (Xia et al., 2021; Xu et al., 2023). Polysaccharides are common natural macromolecules composed of covalently linked monosaccharides, forming different polymer structures with biological activities such as anti-tumor, antioxidant, immune protein regulation, enhancing dendritic cell activity, and cytokine release, which may protect the colon (Wu et al., 2022). Anthraquinone emodin has anti-inflammatory and regulatory effects on intestinal immune symbiosis (Cai et al., 2023; Wang et al., 2023). SCFAs are a series of metabolites produced by the gut microbiota, which have different effects on microbial composition, diversity, and immune system (Tan et al., 2023). Probiotics may be an important therapeutic agent that can be used not only as a single drug, but also as an adjunct to conventional treatments (Zhao et al., 2022).

The most widely used probiotics currently are *Bifidobacterium* and *Lactobacillus*. Lactic acid bacteria are safe microorganisms, which can adjust the microbial community, inhibit cancer, have anti diabetes, anti hyperlipidemia and anti-colitis effects, and induce non-specific activation of the host immune system (Peran et al., 2007). Probiotics ameliorated colitis severity through the inhibition of NF-κB DNA binding activity, a reduction in leukocyte accumulation, and the downregulation of IL-6 and TNF-α expression (Hegazy and El-Bedewy, 2010). It has been demonstrated that *Lactobacillus suntoryeus* HY7801improves colitis through the suppression of LPS-triggered TLR-4 activation, thereby inhibiting downstream NF-κB signal transduction (Lee et al., 2009). The most dynamic microbiome targeted therapy is fecal microbiota transplantation (FMT), which can affect various disease states (Teigen et al., 2025). FMT is effective in treating IBD with recurrent *Clostridioides difficile* infection, and studies have shown that premature use of antibiotics should be avoided (Burdette et al., 2025). FMT induces a persistent increase in microbial diversity and enriches the phylogenetic diversity of gut microbiota in recipients (Paramsothy et al., 2017). Prebiotics, defined as indigestible dietary components, selectively stimulate the proliferation of beneficial gut microbiota, thereby conferring health advantages to the host (Noguera-Fernández et al., 2024). The synergistic interaction between probiotics and prebiotics in synbiotics contributes to improved immune function and gastrointestinal homeostasis. Postbiotics, the bioactive compounds generated during probiotic fermentation, modulate metabolite synthesis, strengthen intestinal barrier integrity, and reshape gut microbiota composition, thereby offering therapeutic potential for metabolic disorders (Al-Habsi et al., 2024).

Diet represents a modifiable environmental factor that influences both the risk of developing IBD and disease severity, while also serving as a potential therapeutic intervention (Sasson et al., 2021; Dryden and Dryden, 2025). A high intake of linoleic acid, animal fats, and sugar is correlated with an elevated risk of IBD. In contrast, diets abundant in fiber and consistent consumption of citrus fruits may confer a protective effect against IBD development (Owczarek et al., 2016).

## 4 Discussion

IBD is a major global health issue, including CD, UC, and IBD-unclassified. IBD pathogenesis involves complex interactions between environmental triggers, immune dysregulation, genetic susceptibility, and gut microbiota alterations. Of particular significance, the intestinal microbiota acts as a key mediator of the complex interplay between environmental, genetic, and immune factors in IBD (Manichanh et al., 2012). IBD patients exhibit significant gut microbiota alterations, characterized by reduced abundance of *Bacteroidetes* and *Firmicutes* alongside increased proportions of *Proteobacteria* and *Actinobacteria* (Sharma et al., 2025).

Dysbiosis of the gut microbiota can directly disrupt local immune homeostasis, manifesting as an imbalance between the functional suppression of Tregs and the abnormal activation of pro-inflammatory Th1/Th17 cells (Neurath, 2014; Round and Mazmanian, 2009). Activated Th1 and Th17 cells secrete a variety of inflammatory cytokines, such as IL-17, IL-21, IL-22, TNF-α and IFN-γ. Among these, excessive IL-17 and IL-21 in the mucosa can induce the expression of MMPs, leading to direct damage to the intestinal epithelial barrier (de Almeida et al., 2022). In contrast, Tregs exert critical immunosuppressive functions by secreting inhibitory factors including TGF-β, IL-10 and IL-35, as well as by regulating the apoptosis of Teffs (Mayne and Williams, 2013). This process is tightly controlled by the transcription factor FOXP3, which is regarded as the master regulator of Treg function (Fontenot and Rudensky, 2005). Notably, the IBD-susceptibility gene *IL23R* encodes the IL-23 receptor, and its signaling pathway plays a central role in the differentiation and maintenance of Th17 cells (Duerr et al., 2006). Meanwhile, IL-23 can further suppress the function of intestinal Tregs, thereby amplifying the inflammatory response and promoting disease progression (Izcue et al., 2008). These findings suggest that targeting the IL-23/Th17 axis may represent a key interventional strategy for restoring immune balance.

Microbial metabolites serve as key mediators in the microbiota-host dialogue. Tryptophan metabolites, such as indole derivatives, function as endogenous ligands for the AHR and can negatively regulate the NF-κB signaling pathway, thereby suppressing excessive inflammatory responses (Jin et al., 2014; Schiering et al., 2017). Additionally, folate metabolism is involved in immune regulation: hyperhomocysteinemia resulting from folate deficiency can enhance the activity of Th17 cells in the intestinal mucosa (Wang et al., 2017), whereas the microbial metabolite IPA promotes apoptosis of Th1/Th17 cells via interaction with HSP70, indicating the bidirectional regulatory role of metabolites on immune cells (Gao et al., 2025).

On the other hand, SCFAs, particularly butyrate, play multifaceted roles in maintaining intestinal homeostasis. In terms of immune regulation, butyrate suppresses NF-κB signaling pathway phosphorylation through a GPR109A-dependent mechanism, thereby alleviating inflammation (Chen et al., 2018a). Regarding the mucosal barrier, butyrate enhances epithelial barrier integrity by activating AMPK to promote tight junction assembly (Peng et al., 2009), upregulating claudin-3 and claudin-4 expression via the Akt signaling pathway (Yan and Ajuwon, 2017), and increasing mitochondrial oxygen consumption in IECs to stabilize HIF and its target genes (Kelly et al., 2015). Meanwhile, SCFAs can also promote intestinal luminal IgA secretion and strengthen mucosal immunity by inhibiting HDACs and activating the mTOR pathway (Smith et al., 2013). Bacterial products regulate the differentiation of Tfh cells and Tfr cells via the TLR-MyD88 signaling axis, driving symbiotic bacteria-specific IgA responses in PP. SCFAs also play a significant regulatory role in this process (Abreu, 2010; Wang et al., 2015).

Genetic factors profoundly influence the host immune response to the gut microbiota and their metabolites. The pattern recognition receptor gene *NOD2* recruits TAK1 and the adaptor proteins TAB1–3 via RIPK2, thereby activating both the NF-κB and MAPK signaling pathways. This activation induces the expression of pro-inflammatory cytokines and AMP-related genes, and dysregulation of this pathway is closely associated with the pathogenesis of IBD (Wang et al., 2001; Ogura et al., 2001; Caruso et al., 2014). Furthermore, inflammasome pathways are also subject to genetic regulation. For instance, microbial metabolites can activate the NLRP6 inflammasome, promoting the production of downstream inflammatory mediators (Levy et al., 2015). Conversely, the protein encoded by the IBD susceptibility gene *IRGM* negatively regulates NLRP3 inflammasome activation by binding to NLRP3 and inhibiting its oligomerization as well as the recruitment of the ASC protein (Mehto et al., 2019). Further research suggests that IRGM may also mediate the degradation of NLRP3 and ASC via a p62-dependent selective autophagy pathway, thereby preventing excessive inflammasome activation (Shi et al., 2012). These findings indicate that genetic background (e.g., variants in *IRGM*) can modulate the intensity of innate immune signaling, thereby influencing host tolerance and reactivity to the intestinal microbial ecosystem and ultimately contributing to IBD pathogenesis.

The onset and progression of IBD represent a networked pathological process shaped by the interplay of gut dysbiosis, metabolic disturbances, immune imbalance, and genetic susceptibility. Microbial metabolites, such as tryptophan derivatives and butyrate, act as critical bridges that deeply participate in maintaining immune homeostasis by modulating key signaling pathways—including those involving AHR and NF-κB—and regulating the balance of T-cell subsets. Meanwhile, genetic factors (e.g., *IL23R*, *NOD2*, and *IRGM*) determine the host’s response threshold to the intestinal milieu by influencing innate immune mechanisms such as receptor signaling and inflammasome activity.

This review synthesizes current knowledge on gut microbiota’s role in IBD, laying the foundation for advancing microbiome research toward clinical applications in diagnosis and treatment. The current evidence base is constrained by limited investigations, leaving several mechanistic aspects incompletely understood. Comprehensive studies are imperative to unravel the complex interplay between hosts and their microbiota, as well as its pathogenic relevance, thereby enabling evidence-based therapeutic approaches.
